# Increased levels of GM-CSF and CXCL10 and low CD8^+^ memory stem T Cell count are markers of immunosenescence and severe COVID-19 in older people

**DOI:** 10.1186/s12979-024-00430-7

**Published:** 2024-05-07

**Authors:** Johanne Poisson, Carine El-Sissy, Arnaud Serret-Larmande, Nikaïa Smith, Morgane Lebraud, Jean-Loup Augy, Catherine Conti, Cécile Gonnin, Benjamin Planquette, Jean-Benoît Arlet, Bertrand Hermann, Bruno Charbit, Jean Pastre, Floriane Devaux, Cyrielle Ladavière, Lydie Lim, Pauline Ober, Johanna Cannovas, Lucie Biard, Marie-Christelle Gulczynski, Noémie Blumenthal, Hélène Péré, Camille Knosp, Alain Gey, Nadine Benhamouda, Juliette Murris, David Veyer, Eric Tartour, Jean-Luc Diehl, Darragh Duffy, Elena Paillaud, Clémence Granier

**Affiliations:** 1 Université Paris Cité, Paris, France; 2 Department of Geriatric Medicine, Hôpital Europeen Georges Pompidou, AP-HP, Paris, France; 3 Inserm U1149, Center for Research on Inflammation, Paris, France; 4 INSERM, Laboratory of Integrative Cancer Immunology, Paris, France; 5 Cordeliers Research Center, Sorbonne University, University Paris Cité, Paris, France; 6 Department of Immunology, APHP, Hôpital Européen Georges Pompidou (HEGP), Paris, France; 7 ECSTRRA Team, UMR-1153, Université Paris Cité, INSERM, AP-HP, Saint Louis Hospital, Paris, France; 8 Translational Immunology Unit, Institut Pasteur, Université Paris Cité, Paris, France; 9 Medical intensive care unit, Hopital Delafontaine, 2 rue du Dr Delafontaine, Saint-Denis, 93200 France; 10 Université Paris Cité, INSERM, PARCC, Paris, France; 11 Service de Pneumologie Et Soins Intensifs, Hôpital Européen Georges Pompidou, AP-HP, Paris, France; 12 Internal Medicine Department, Georges Pompidou European Hospital, Assistance Publique-Hôpitaux de Paris (AP-HP), Paris, France; 13 Faculty of Medicine, Université Paris Cité, Paris, 75006 France; 14 Medical Intensive Care Unit, AP-HP. Centre Université Paris Cité, Georges Pompidou European Hospital, Paris, 75015 France; 15 INSERM UMR 1266, Institut de Psychiatrie Et Neurosciences de Paris (IPNP), Université Paris Cité, Paris, France; 16 Institute of Ophthalmology, University College London (UCL), London, UK; 17 Gérontologie 1, GHU AP-HP. Centre Université Paris Cité, Corentin Celton Hospital, Issy-Les-Moulineaux, 92130 France; 18 Virology Laboratory, Hôpital Européen Georges-Pompidou, APHP.Centre - Université Paris Cité, Paris, France; 19 Centre de Recherche Des Cordeliers, Sorbonne Université, Inserm, Université de Paris, Functional Genomics of Solid Tumors Laboratory, Équipe Labellisée Ligue Nationale Contre Le Cancer, Labex OncoImmunology, Paris, France; 20 Université Paris Cité, Faculté de Santé, UFR de Médecine, Paris, France; 21 HeKA, Inria Paris, Inserm, Université Paris Cité, Paris, France; 22 University Paris Cité, Innovative Therapies in Hemostasis, INSERM, Paris, 75006 France; 23 Univ. Paris Est Créteil, Inserm U955, IMRB, Créteil, France

**Keywords:** Immunosenescence, Inflammaging, Immune-ageing, COVID-19, Geriatrics, Infections, Senescence-associated secretory phenotype, CD8-positive T-lymphocytes, GMCSF, CXCL10, TSCM

## Abstract

**Background:**

Ageing leads to altered immune responses, resulting in higher susceptibility to certain infections in the elderly. Immune ageing is a heterogeneous process also associated with inflammaging, a low-grade chronic inflammation. Altered cytotoxic T cell responses and cytokine storm have previously been described in severe COVID-19 cases, however the parameters responsible for such immune response failures are not well known. The aim of our study was to characterize CD8^+^ T cells and cytokines associated with ageing, in a cohort of patients aged over 70 years stratified by COVID-19 severity.

**Results:**

One hundred and four patients were included in the study. We found that, in older people, COVID-19 severity was associated with (i) higher level of GM-CSF, CXCL10 (IP-10), VEGF, IL-1β, CCL2 (MCP-1) and the neutrophil to lymphocyte ratio (NLR), (ii) increased terminally differentiated CD8^+^T cells, and (ii) decreased early precursors CD8^+^ T stem cell-like memory cells (TSCM) and CD27^+^CD28^+^. The cytokines mentioned above were found at higher concentrations in the COVID-19^+^ older cohort compared to a younger cohort in which they were not associated with disease severity.

**Conclusions:**

Our results highlight the particular importance of the myeloid lineage in COVID-19 severity among older people. As GM-CSF and CXCL10 were not associated with COVID-19 severity in younger patients, they may represent disease severity specific markers of ageing and should be considered in older people care.

**Supplementary Information:**

The online version contains supplementary material available at 10.1186/s12979-024-00430-7.

## Background

The recent COVID-19 pandemic highlighted the role of ageing as a risk factor for severe disease after infection. Indeed, age has been described as an independent risk factor for both severity and mortality during COVID-19 [1, 2]. Ageing leads to modifications in adaptive immunity with thymic involution and reduction of naive T cells while memory and differentiated T cells (i.e. CD27^−^CD28^−^ and EMRA) accumulate [3].

CD8^+^ T cells are the main cytotoxic effectors and are major players of antiviral defence through their killing activity of infected cells after antigen recognition [4]. Ageing is accompanied by a CD4 and CD8 lymphopenia, and a decrease of both CD8^+^ T cell priming and generation after a newly encountered antigen [5]. This decreased specific CD8^+^ T cell response may be responsible for poor vaccine efficacy and may lead to severe manifestations from seasonal infection or latent virus reactivation in some individuals [6]. For instance, both Th1 response and CD8^+^ T cell cytotoxicity are impaired following an in vitro influenza virus exposure in older population [7]. After SARS-CoV-2 infection, T cells confer protective immunity [8] and CD8^+^ T cell memory persist up to 6 to 8 months in half of the affected individuals [9]. A coordinated adaptive immune response is protective against severe forms of COVID-19 [1]. In older COVID-19^+^ patients, there is (i) an impaired capacity of CD8^+^ T cell priming after in vitro stimulation [10] and (ii) less secretion of granzyme A and B by CD8^+^ T cells after SARS-CoV2 infection than in younger patients [11].

Biomarkers of T cell senescence have emerged recently. CD8^+^ T cells in particular, can overexpress (i) receptors usually found on natural killer (NK) cells such as CD57, KLRG1, CD56 and NKG2a and (ii) a Sestrin-2/MAP-Kinases complex [3, 12]. SARS-CoV-2 infection has been associated with higher and persistent CD57 expression by CD8^+^ T cells [13], but a characterization of the CD8 senescence profile with all the above-mentioned markers has not been performed.

To our knowledge, the association between immune ageing and COVID-19 in older population has not been reported yet.

The dysregulated immune response observed in severe COVID-19 cases could also be partly due to a non-specific inflammation [1, 14]. In COVID-19, a cytokine storm is involved in the severe cases and is in part responsible for tissue lesions particularly in lungs [15]. Ageing can be accompanied by inflammaging which is a low-grade chronic inflammation that is observed in healthy individuals even without any infection. This inflammation is characterized by an elevation of C reactive protein (CRP) and cytokines such as IL-6, IL-1β, CCL2 (MCP-1), CXCL8 (IL-8) and CXCL1 (GRO-A) even in the absence of any detectable stimulus [3, 16]. Additionally, some cytokines such as IL-1Ra and IL-10 seem to display anti-inflammaging properties and may favour longevity [17]. These cytokines are also produced by senescent non-lymphoid cells comprising the senescence-associated secretory phenotype (SASP). The SASP is a secretome of cytokines including the ones mentioned above as well as TNFα, IL-1Ra, CCL20 (MIP-3A), CXCL-2 (GRO-B), GM-CSF, G-CSF and VEGF [16]. It is interesting to note that all these cytokines associated with inflammaging and SASP favour the myeloid response (i.e. myelopoiesis and cell recruitment and proliferation).

Besides the lymphoid response which is protective, the myeloid response (neutrophils and monocytes) is also implicated in disease severity and tissue lesions during COVID-19 [18–20]. Neutrophil and macrophage circulating biomarkers are associated with mortality in older patients [21].

Thus, in addition to the CD8 senescence profile, we also characterized the inflammaging and myeloid lineage with (i) other cytokines involved in myeloid cell migration, activation or differentiation such as G-CSF, IL-33, fractalkine or others secreted by myeloid cells such as CXCL10 (IP-10) and (ii) neutrophil and monocyte cell counts.

In this study, we performed comprehensive immune phenotyping in older patients during COVID-19, by exploring the cytotoxic T cell senescent phenotype and soluble inflammaging markers. We compared hospitalized patients over 70 years old affected (COVID-19^+^ cohort stratified on severity) or not (control cohort). A younger cohort was used to compare the age-differential positive association of cytokines with severity. We aimed to better characterize CD8^+^ T cells and cytokines factors that are associated with COVID-19 severity in older patients.

## Material and methods

### Study design

Hospitalized patients were prospectively enrolled from 24/04/2020 to 19/03/2021 in the Georges Pompidou European Hospital (HEGP), Paris, France during the first and second COVID-19 waves. The local Ethical Committee approved this study (CERAPHP “Centre Comité d'éthique de la recherche AP-HP Centre”, IRB registration: #00011928). One hundred and four patients were included in the study: 81 patients in the COVID-19^+^ group and 23 in the control group (COVID-19 negative).

Inclusion criteria in the COVID-19^+^ group were as follows: > 70 years old, confirmed SARS-CoV-2 infection (positive RT-PCR test on a respiratory sample: nasopharyngeal swab or invasive respiratory sample), initial onset of COVID-19 signs/symptoms within 8 days prior to the day of inclusion.

A comparative control group matched on age and sex was constituted within the same period including 23 patients negative for COVID-19, hospitalized in the geriatric department for non-infectious diseases. Exclusion criteria for controls were confirmed or active infection and a positive SARS-CoV-2 RT-PCR test.

In the whole cohort, exclusion criteria were the presence of hemopathy or a treatment by long-term immunosuppressive drug.

A flow chart illustrates the study design (Figure S1).

### Data and sample collection

Sociodemographic characteristics (age, gender, housing: home or nursing home), previous medical history, daily treatment, clinical symptoms in the COVID-19^+^ cohort, corticoid treatment introduced during the COVID-19 care, in-hospital mortality and laboratory data were collected and extracted from a secured and standardized electronic case report form (eCRF Redcap).

Routine blood tests, including complete blood counts (CBC), plasmatic biochemical tests (including markers of renal and liver function, lactate dehydrogenase (LDH) and electrolytes), C-reactive protein (CRP), ultrasensitive I-troponin were collected.

Blood samples were drawn as soon as the COVID-19 diagnosis was confirmed and sent the same day to our laboratory for immunophenotyping and cytokines measurement. Thus, biological samples were taken before any COVID-19 treatment (including dexamethasone) was started.

Comorbidity severity was assessed with the Charlson Comorbidity Index (CCI) [22] and frailty was assessed with the adjusted Rockwood Frailty Scale (RFS) [23].

The time of COVID-19 onset was defined as the date when the first signs or symptoms were noticed. Signs or symptoms included fever, shortness of breath, increased respiratory rate, dry cough, chest tightness, fatigue, myalgia, hypotension, pharyngalgia, diarrhea, nausea, vomiting, abdominal pain, dizziness, delirium, headache, anosmia and agueusia. A quick Sequential Organ Failure Assessment (qSOFA) score was calculated for COVID-19^+^ patients [24]. The qSOFA score ranges from 0 (best) to 3 (worst) points, with one point allocated for: (1) systolic blood pressure ≤ 100 mm Hg, (2) respiratory rate ≥ 22 breaths/min (3) altered mental status (Glasgow Coma Score < 14).

### *COVID-19*^+^*groups stratification based on severity*

Patients included in the COVID-19^+^ groups were classified, in the first 48 h following diagnosis, according to the WHO Clinical Progression Scale (0–10) [25] adapted to the elderly population, then stratified into 3 groups:

- Group 1: Non-severe COVID-19 defined as asymptomatic or symptomatic without oxygen requirement (WHO score range 2–3).
- Group 2: Non-critical COVID-19 defined as median oxygen requirement (≥ 3L/min) with nasal or mask prongs (WHO score range 4–5).
- Group 3: Critical COVID-19 defined as high flow oxygen therapy (HFOT) or Non-Invasive Ventilation (NIV) or Invasive Mechanical Ventilation (IMV) (WHO score range 6–9).

Being a real-life geriatric cohort with a lot of patients with multiple comorbidities and loss of autonomy, some patients in group 3, who developed a severe form of COVID-19 and had a theorical indication to be admitted to an intensive care unit, were not, based on multidisciplinary decision. Indeed, the benefit of such admission was collegially considered too minimal by the physicians. Such decision was taken in accordance with the family and the wishes of the patient when possible. They were nevertheless classified in the COVID-19 critical group (group 3).

### T cell immunophenotype by flow cytometry

Fresh whole blood (100 µL) was labelled for surface and intracellular markers distributed in 3 flow cytometry panels: panel 1: CD3, CD8, CD4, CD45RA, CD27, CD28 and CD95, panel 2: CD3, CD8, CD4, CD45RA, CD27, CD28, Ki-67 and Sestrin-2 and panel 3: CD3, CD8, CD4, CD56, CD57, NKG2A, KLRG1 and DAP12 (see Table S4 for the specific clones and brand). All were direct stainings except for Sestrin-2. All stainings were performed according to the manufacturer’s recommendations, with Sestrin-2 expression being assessed after fixation and permeabilization (Biolegend FoxP3 Perm and Fix). After labelling, red blood cells were lysed using Versalyse lysing solution (Beckman Coulter, Inc., USA) according to the manufacturer’s recommendations. Surface and intracellular markers were analysed by flow cytometry using a Navios® flow cytometer (Beckman Coulter, Inc., USA) according to the manufacturer’s recommendations. Flow set and Flow-check fluorosphere (Beckman Coulter, Inc., USA) were used to calibrate our cytometer on days of experiment. Fluorescence minus one (FMO) controls were used to verify the absence of spillover after applying the compensation matrix and as gating controls. Additionally, isotype controls were used for Sestrin-2, Dap-12, Ki-67, NKG2A and CD95.

### *Characterization of CD8*^+^*T cell differentiation and senescence by flow cytometry*

As highly differentiated memory T cells accumulate with ageing, we studied T cell differentiation. We used CD45RA, CD27 and CD28 cell surface markers to define: naive N (CD45RA^+^CD27^+^), central memory CM (CD27^+^CD45RA^−^), effector memory EM (CD45RA^−^CD27^−^) and EMRA (CD45RA^+^CD27^−^) T cells [12]. We used CD95 to discriminate stem-like memory T cell TSCM from naive T cells [26]. Thus, TSCM were defined as CD45RA^−^ CD27^+^ CD28^+^ CD95^+^ [13, 26] (Figure S2). As initially described by Lugli et al., TSCM CD95 expression levels were lower compared with the CM CD95 expression levels on CD8 (*p* < 0.0001 Figure S2) [26]. As NK receptors and Sestrins accumulate in differentiated T cells [12], we studied CD56, CD57, NKG2A, KLRG1 and Sestrin-2 expression. We characterized the CD8 senescence profile of each patient according to the percentage of EMRA and CD27^−^CD28^−^ subsets, percentage of CD56, CD57, KLRG1 and NKG2A expression and Sestrin-2 expression level in mean fluorescence intensity (MFI) normalized on control isotype (nMFI). The gating strategy is shown in Figure S2.

### Cytokine assays

Plasma samples were put on ice and collected as soon as possible after arrival in the lab. Before protein analysis, plasma and nasal samples were treated in a P3 laboratory for viral decontamination using a protocol previously described for SARS-CoV, which we validated for SARS-CoV-2 [14]. Briefly, samples were treated with 1% TRITON X100 (vol/vol) and 0.3% tri-N-butyl phosphate (vol/vol) for 2 h at room temperature. Tri-N-butyl phosphate was removed before cytokine analysis by passing the treated samples though C18 columns. IL-6, TNF and IL-10 were measured with a commercial triplex assay (Quanterix) on a Simoa HD-1 analyzer (Quanterix). An additional 38 cytokines and chemokines were measured in plasma supernatants with a commercial Luminex multi-analyte assay (Biotechne, R&D systems). Data were acquired on a Bio-Plex 200 System (Bio-Rad) and analyzed with Bio-Plex Manager v5 (Bio-Rad).

### Statistical methods

The significance of associations between the severity groups and biological or clinical variables was assessed using either t-test or ANOVA for continuous variables, and likelihood-ratio test of logistic regression coefficients for categorical ones. For each association, statistical tests were computed both in univariate and bivariate settings, with the latter incorporating age into a multivariable model. This inclusion of age was essential due to the notable age difference between groups and its potential association with various biological and clinical markers under study. Correlations between the different biological markers was asserted using Pearson correlation coefficient (presented as correlation matrices). The threshold for statistical significance was set at 5%.

Furthermore, Principal Component Analysis (PCA) followed by k-means clustering were employed to investigate (i) the association between severity groups and CD8^+^ T cell senescence, and (ii) the association between severity groups and cytokines. A PCA was carried-out in a first step, and the individual patients were then grouped together using k-means based on the first two-dimensions of the PCA.

A Manhattan plot summarizes the statistical associations for the variables pertaining to either CD8^+^ T cell immunosenesence, or clinical group. The 5% statistical significance threshold was corrected on this figure to account for the multiple testing using the False Discovery Rate method.

### *External dataset of independent younger COVID-19*^+^*cohort*

For the cytokine dosage, we compared our data set (median age of the current cohort: 86 years old) with an independent younger cohort (median age of 55 years old) already described and published using the same method of dosage by the same lab [14]. The dosage were performed by the same operator. We chose the closest timepoint from the symptom onset and the closest degree of COVID-19 severity for the comparisons (group 2 and 3).

## Results

### Characteristics of the cohort (Table 1)

**Table 1:** Demographic and baseline characteristics of the control and COVID19 + cohorts stratified by clinical severity

	Controls (*N* = 23)	Group 1 (*N* = 32)	Group 2 (*N* = 20)	Group 3 (*N* = 29)	Total (*N* = 104)	*p*-value
**Sexe**						0.018
Female	15 (65.2%)	23 (71.9%)	13 (65.0%)	10 (34.5%)	61 (58.7%)	
Male	8 (34.8%)	9 (28.1%)	7 (35.0%)	19 (65.5%)	43 (41.3%)	
**Age (years)**						< 0.001
Mean (SD)	85.58 (6.49)	85.40 (5.97)	88.80 (5.25)	81.02 (6.21)	84.87 (6.53)	
**Body mass index (BMI)**						0.007
Mean (SD)	23.22 (5.31)	22.70 (4.73)	25.40 (4.20)	27.04 (4.92)	24.51 (5.09)	
**Chronic lung disease**						0.858
	4 (17.4%)	4 (12.5%)	2 (10.0%)	5 (17.2%)	15 (14.4%)	
**Diabetes**						0.440
	1 (4.3%)	6 (18.8%)	2 (10.0%)	4 (13.8%)	13 (12.5%)	
**Hypertension**						0.183
	11 (47.8%)	23 (71.9%)	10 (50.0%)	19 (67.9%)	63 (61.2%)	
**Cardiac history**						0.442
	17 (73.9%)	19 (59.4%)	12 (60.0%)	15 (51.7%)	63 (60.6%)	
**Charlson comorbidity index**						0.198
Mean (SD)	4.65 (2.31)	5.19 (2.76)	5.00 (2.68)	3.79 (2.50)	4.62 (2.59)	
**Rockwood frailty index**						< 0.001
Mean (SD)	4.87 (1.69)	5.39 (1.45)	5.00 (1.37)	3.50 (1.97)	4.67 (1.80)	
**qSOFA**						NA
Mean (SD)	NA	0.31 (0.69)	0.85 (0.59)	1.17 (0.80)	0.75 (0.80)	
**Desaturation < 90%**						NA
	0	0 (0.0%)	11 (55.0%)	23 (79.3%)	34 (42.0%)	
**Dexamethasone (COVID-19 treatment)**						< 0.001
	0 (0.0%)	1 (3.1%)	9 (45.0%)	23 (79.3%)	33 (31.7%)	
**Corticotherapy (usual treatment)**						0.416
	0 (0.0%)	1 (3.1%)	2 (10.0%)	2 (7.1%)	5 (4.9%)	
**Serotonin reuptake inhibitor (usual treatment)**						0.005
	2 (9.1%)	14 (43.8%)	5 (26.3%)	3 (10.3%)	24 (23.5%)	
**Death**						< 0.001
	0 (0.0%)	0 (0.0%)	0 (0.0%)	17 (58.6%)	17 (20.7%)	

Patients included in the COVID-19 cohort were classified according to the WHO Clinical Progression Scale (0–10) [25] adapted for the elderly population in (i) group 1 (non-severe COVID-19) for asymptomatic or symptomatic without oxygen requirement (WHO score range 2–3), (ii) group 2 (non-critical COVID-19) if median oxygen requirement ≥ 3L/min (WHO score range 4–5) and (iii) group 3 (critical COVID-19) when theoretically requiring high flow oxygen therapy (*HFOT*, *NIV* Non-Invasive Ventilation, *IMV* Invasive Mechanical Ventilation) (WHO score range 6–9)

Cardiac history includes atrial fibrillation, coronary artery disease, heart failure, obliterating arteriopathy of the lower limbs, valve disease and stroke. The group 3 was younger compared with the other groups. The BMI was higher in the group 3 compared with the other groups. Dexamethasone was used mainly in the moderate to severe cases. Death events occurs only in the group 3, by definition. The frailty index was lower in the group 3 compared to the other groups. The comorbidities (pulmonary, cardiac, hypertension and diabetes) and the Charlson comorbidity index were not different among the different groups. The critical group and the control cohort received less serotonin reuptake inhibitor in usual treatment compared to the non-severe/non critical COVID-19^+^ group. The significance of associations between severity groups and biological and clinical variables was determined using multiple ANOVA for continuous covariates

One hundred and four in-patients were consecutively included in the prospective study conducted in Georges Pompidou European hospital: (i) 81 COVID-19^+^ patients including 27 patients hospitalized in intensive care unit (ICU) and 54 in the geriatric department (non-ICU) and (ii) 23 patients in the control group (SARS-CoV-2 negative).

The median age of the overall cohort was 86.2 years old [70.8–98.3]. COVID-19^+^ and control cohorts were matched for age and sex. The main characteristics of the population are summarized in Table 1. Patients in the critical COVID-19^+^ group (group 3) were younger compared to the other groups.

As described in Table 1, the male/female ratio and Body Mass Index (BMI) were significantly higher in the critical COVID-19^+^ group (group 3) compared to the other groups. Group 3 patients received less serotonin reuptake inhibitor antidepressant drugs. Comorbidities such as lung and cardiac chronical pathology, hypertension and diabetes were not associated with COVID-19 severity. Consistently, no difference in the Charlson Comorbidity Index was observed. The Rockwood Frailty Index was higher in groups 1 and 2 (non-severe and non-critical group, respectively) compared with group 3 (critical group) and the control group. Death events were observed only in the severity group 3. Dexamethasone as medical care treatment for COVID-19 was recommended by the medical authorities during the study. As expected, mainly severity group 2 and 3 patients benefited from it. Only 5 patients received corticoids for indications other than COVID-19 (no statistical difference between groups).

### Immune markers associated with COVID-19 in older people

In order to investigate the modifications that occurred during SARS-CoV-2 infection in older patients, the COVID-19^+^ cohort was compared with the control cohort. The results are presented as means ± standard deviations.

#### CD8^+^ T cell senescence in the COVID 19^+^ compared to the COVID 19^−^ cohort (Fig. 1)

**Fig. 1:**
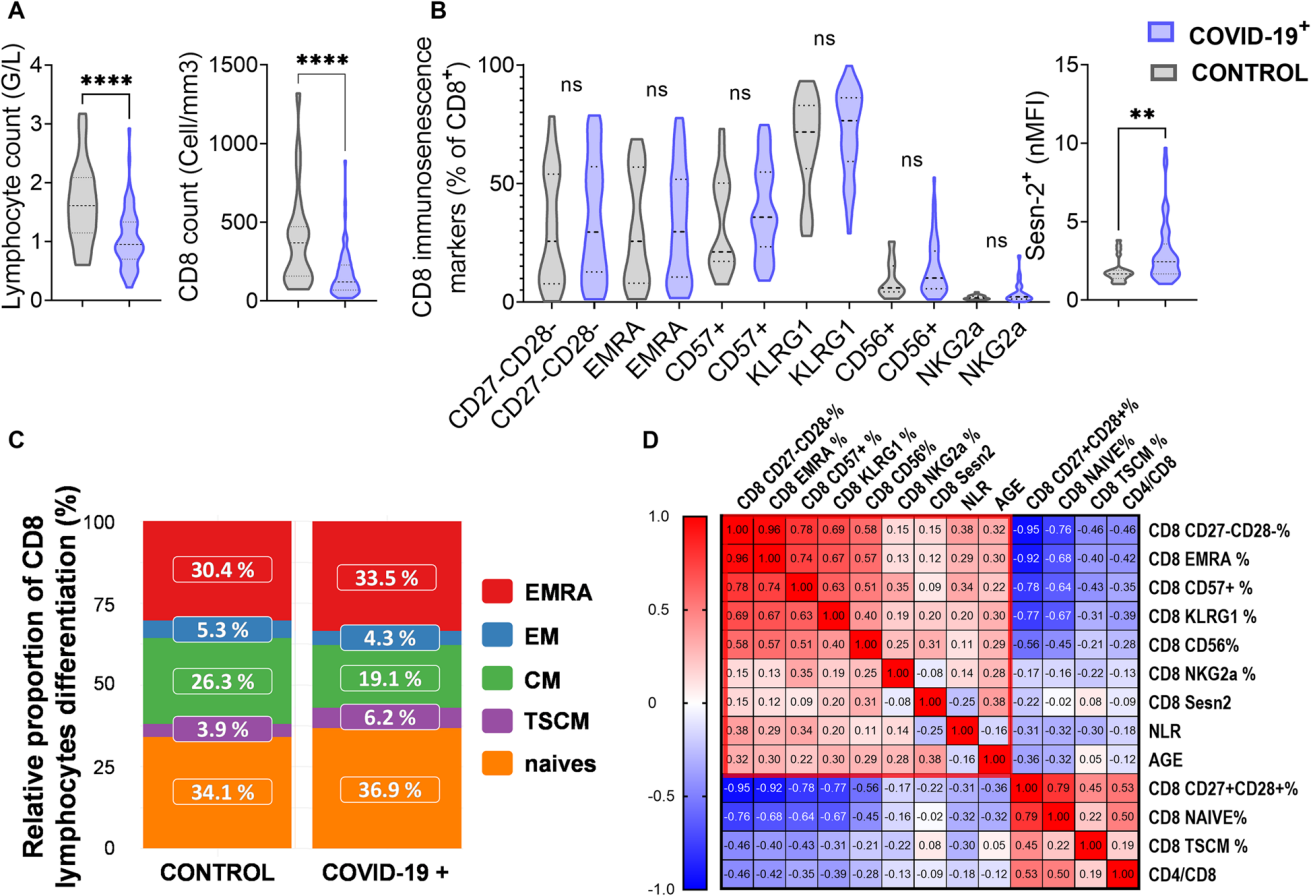
CD8^+^ T cell senescence and COVID-19 infection. The COVID-19^+^ cohort (blue violin plot) was compared with the control cohort (grey violin plot) for the following parameters: (A) Lymphocyte and CD8^+^ T cell counts, (B) CD8 senescence markers characterized by percentage of CD27^−^CD28^−^, EMRA, CD57^+^, KLRG1^+^, CD56^+^, NKG2a^+^ among CD8^+^ T cell and normalized mean fluorescence intensity of Sestrine-2 (Sesn-2) expression in CD8^+^ T cell, (C) CD8^+^ T cell differentiation stages i.e. naïve, T stem cell memory (TSCM), effector memory (EM), central memory (CM) and EMRA percentages among CD8 + T cells and (D) A correlation matrix of these parameters. A red rectangle denotes the CD8 senescence profile. Two-group differences were tested using a two-sided unpaired t-test. The difference was considered as significant when the *p*-value was equal to or under 0.05. The *p*-values are summarized with asterisks (< 0.05*, < 0.01**, < 0.001***, < 0.0001****, ns non significant)

The COVID-19^+^ patients had significantly lower levels of total lymphocytes (1.0 ± 0.1 *versus* 1.6 ± 0.7 G/L in the control group, *p* < 0.0001) and CD8^+^ T cells (170.3 ± 63.8 *versus* 389.4/mm^3^ ± 312.1 in the control group, *p* < 0.0001) (Fig. 1A). The CD4/CD8 ratio was not statistically different between the two groups (4.0 ± 3.1 *versus* 3.6 ± 2.5 in the COVID-19^+^ and control group respectively, *p* = ns).

The percentage of CD57, KLRG1, CD56, NKG2A expressed by CD8^+^ T cells and late differentiated CD27-CD28-, EMRA CD8 subsets were not significantly different between the COVID-19^+^ and control cohort (Fig. 1B). In both cohorts, KLRG1 was highly expressed among CD8^+^ T cells (71.2% ± 2.7 in COVID-19^+^ *versus* 67.5% ± 19.8 in control group, *p* = ns) followed by CD57 (40.2% ± 1.6% *versus* 32.5% ± 20.2, *p* = ns), whereas CD56 and NKG2A were weakly if not expressed (14.5% ± 2.1 *versus* 9.3% ± 7.6 and 4.1%, SD: 1.1% *versus* 2% ± 0.9 respectively, *p* = ns). The percentage of the late differentiated CD8^+^ T cells CD27^−^CD28^−^ and EMRA subsets were respectively 35.2% ± 2.6 *versus* 31.5% ± 24.4 and 33.7 ± 2.1 *versus* 30.3% ± 23.4 (*p* = ns). CD8^+^ T cell expression of Sestrin-2 was significantly higher in the COVID-19^+^ group (nMFI = 3.01 ± 1.92 *versus* 1.89 ± 0.77 in the control group, *p* = 0.034 Fig. 1B).

The central memory (CM) population tended to be decreased in absolute count and percentage among CD8^+^ T cells in the COVID-19^+^ compared with the control cohort ( *p* = 0.056) while naive, TSCM, and EM CD8^+^ T cell subsets were not significantly different (Fig. 1C and Table S1). A lower count of naïve and EM cells, but not TSCM cells, were observed in COVID-19^+^ patients (Table S1).

Finally, a correlation matrix with the above-mentioned CD8 senescence-related parameters was generated to identify a relevant pattern (Fig. 1D). The CD27^−^CD28^−^, EMRA, CD57, KLRG1 and CD56 percentages among CD8^+^ T cells were significantly positively correlated together (*p* < 0.05). Sestrin-2 expression, CD56%, CD27^−^CD28^−^% and KLRG1% were positively associated with age (*p* < 0.05). These parameters could therefore define a CD8 senescence profile. The Neutrophil to Lymphocyte Ratio (NLR), usually associated with immunosenescence [27], was positively correlated with CD27^−^CD28^−^, EMRA and CD57 percentages among CD8. The percentages of earlier CD8^+^ T cell stages CD27^+^CD28^+^, naïve and TSCM, usually in higher proportion in younger people [28], were inversely correlated with the CD8 senescence profile as did the CD4/CD8 ratio (Fig. 1D).

#### Inflammaging and myeloid lineage compared to the control cohort and to a younger COVID-19^+^ cohort

Cytokines previously described in the literature as associated with senescence (TNFα, IL-6, VEGF, IL-1RA, GM-CSF, CCL20, CXCL8, IL-1β, CXCL1, CXCL2, G-CSF, CCL2 and IL-10) [16, 17, 29, 30] or with the recruitment of myeloid cells (CXCL10, fractalkine and IL-33) were compared between COVID-19^+^ and control cohorts. Concentrations were significantly higher in the COVID-19^+^ cohort except for IL-1β, CXCL1, IL-10 and CXCL8 (Table S2). To note, CXCL8 concentrations were considered too low to be interpretable (< 10µg/mL).

To validate the association of these cytokines with senescence during COVID-19 infection, the data set from our cohort (median age of 86 years old) was compared with an independent external dataset of younger patients (median age of 55 years old) for which cytokine concentrations were measured with the same method performed by the same operator in the same laboratory [14]. During COVID-19 infection, TNFα, VEGF, GM-CSF, IL-1β, CXCL10, fractalkine, IL-33 and CCL2 were significantly at higher concentrations in older than younger patients. Conversely, plasma IL-10, CXCL1 and CXCL2 concentrations were significantly lower in older patients compared to younger patients. Plasma IL-6, IL-1Ra, CCL20, CXCL8 and GCSF were found at similar concentrations in young and old patients (Table 2) [14].

**Table 2:** cytokines in COVID-19^+^: older *versus* younger patients

Variable in mean (SD) pg/mL	COVID-19^+^ older patients cohort (median age 86 [70–98])	Younger independant cohort (*N. Smith *et al. *2021* COVID-19^+^Adult median age 55 [25–79])	*p*-value
**plasma TNFa**	54.7 (26.7)	16.4 ( 9.6)	< 0.001
**plasma VEGF**	441.4 (308.2)	227.9 (185.4)	< 0.001
**plasma GMCSF**	99.1 (56.7)	17.1 (11.0)	< 0.001
**plasma IL-1B**	15.9 (7.1)	3.1 (0.5)	< 0.001
**plasma FRACTALKINE**	1711.0 (1368.0)	252.1 (115.6)	< 0.001
**plasma IL-33**	31.6 (13.4)	14.0 (15.2)	< 0.001
**plasma CXCL10**	522.8 (725.3)	94.8 (207.5)	0.002
**plasma CCL2**	428.8 (415.0)	222.1 (335.2)	0.017
plasma IL-6	888.7 (4638.9)	121.3 (444.6)	ns
plasma IL-1RA	4728.6 (6890.7)	2429.8 (2129.1)	ns
plasma CCL20	137.9 (246.2)	87.9 (159.4)	ns
plasma CXCL8	18.9 (36.3)	8.0 (21.2)	ns
plasma GCSF	89.0 (125.2)	88.9 (101.6)	ns
***plasma IL-10***	287.3 (297.6)	2049.6 (1341.1)	< 0.001
***plasma CXCL1***	145.0 (113.9)	958.5 (574.1)	< 0.001
***plasma CXCL2***	303.5 (350.2)	917.7 (1676.0)	0.005

The COVID-19^+^ older cohort was compared with a younger independent cohort for senescence associated cytokine plasma concentration (same lab & method & operator). Cytokines in italic bold were in lower concentrations whereas cytokines in bold were in higher concentrations in the elderly. For others, no difference between the young and elderly was observed. *P*-value is from two-sided unpaired t-test. The difference was considered as significant when the *p*-value was egal or under 0.05

The neutrophil count was significantly higher in the COVID-19^+^ compared to the control cohort (6.8 ± 2.4 and 3,7 ± 1.5 G/L respectively, Figure S3). The NLR was also significantly increased in the COVID-19^+^ compared to the control cohort (10.1 ± 8.1 and 3.1 ± 2.7 respectively, Figure S3). No significant differences between leucocyte and monocyte counts were observed (8.5 ± 5.8 *versus* 6.8 ± 2.2 and 0.6 ± 0.1 *versus* 0.6 ± 0.1G/L respectively, Figure S3).

The association between senescent and myeloid cytokines and cells was assessed. In a correlation matrix, TNFα, VEGF, GM-CSF, IL-1β, CXCL10, CCL2, IL-6, CCL20, G-CSF, IL-10, IL-1RA, NLR, neutrophil and monocyte counts positively clustered together and could therefore define a “myelo-senescence” profile. CRP, an inflammation marker increased in inflammaging [30], positively correlated with this profile. GROA/B, fractalkine and IL-33 were not associated with this cluster (Figure S4). Finally, no correlation between the CD8 senescence and myelo-senescence profiles was found (data not shown).

### Immune ageing markers associated with COVID-19 severity in older people

#### CD8 senescence profile

When comparing the 3 groups, the critical COVID-19^+^ patients (group 3) displayed a significantly more severe CD8 lymphopenia than non-critical patients (groups 1 and 2). The percentages of senescent CD8^+^ T cells i.e. CD27^−^CD28^−^, EMRA and CD57 among CD8^+^ T cells were significantly increased in the severity groups 2 and 3 after adjustment for age (Table 3). To note, these associations were not found when considering the absolute values of these parameters. Sestrin-2 expression by CD8^+^ T cells was only positively associated with increased severity without adjustment for age. KLRG1 and CD56 expression on CD8 were not associated with severity. The percentage and number of early differentiated CD8^+^ T cells i.e. TSCM-like and CD27^+^CD28^+^ CD8^+^ T cells were significantly lower in the severity groups 2 and 3 compared to group 1 (Table 3 and Figure S5). To note, this difference was observed in absolute numbers for naïve CD8^+^ T cells, but not in percentage (Table 3 and Table S1). When comparing CD8 differentiation i.e. EM, EMRA and CM between severity groups, no difference was observed in both numbers and percentages (Table S1).

**Table 3:** CD8-senescence association with severity

Variable	Unit	**Group 1**		**Group 2**		**Group 3**		Univariate *p*.value	Adjusted *p*.value
		mean	SD	mean	SD	mean	SD		
CD8^+^	(/mm3)	198.8	(132.9)	212.4	(201.0)	99.9	(73.6)	**0.006**	**0.046**
**LT-CD8 senescence**									
CD57^+^	(% CD8)	31.9	(16.9)	46.3	(19.9)	42.5	(19.0)	**0.047**	**0.036**
	(/mm^3^)	70.8	(83.1)	102.8	(143.8)	40.9	(30.9)	0.161	0.435
CD27^**−**^ CD28^**−**^	(% CD8)	26.3	(22.4)	40.8	(23.3)	38.5	(27.3)	0.105	**0.037**
	(/mm^3^)	64.2	(91.1)	97.5	(113.2)	37.8	(33.7)	0.110	0.477
EMRA	(% CD8)	26.2	(21.7)	37.6	(21.8)	37.2	(25.3)	0.166	**0.054**
	(/mm^3^)	62.6	(83.2)	81.3	(76.2)	36.2	(30.7)	0.138	0.565
KLRG1^+^	(% CD8)	69.8	(16.6)	75.8	(18.6)	67.8	(21.9)	0.482	0.939
	(/mm^3^)	149.3	(131.6)	167.5	(204.7)	63.2	(48.1)	0.073	0.192
CD56^+^	(% CD8)	13.0	(9.4)	18.4	(13.5)	12.3	(12.3)	0.275	0.680
	(/mm^3^)	30.1	(38.3)	50.7	(99.5)	10.2	(8.9)	0.121	0.365
Sestrin-2	nMFI/CD8	2.9	(1.2)	4.1	(2.9)	2.3	(1.4)	***0.013***	0.219
DAP12	nMFI/CD8	14.2	(25.0)	18.4	(23.9)	14.1	(7.9)	0.802	0.887
**LT-CD8 early stages**									
Naive	(% CD8)	38.8	(16.0)	32.4	(20.0)	37.7	(18.1)	0.551	0.571
	(/mm^3^)	70.1	(34.6)	53.9	(54.6)	39.0	(34.7)	**0.045**	**0.030**
TSCM	(% CD8)	8.6	(7.0)	5.6	(3.8)	3.7	(2.6)	**0.009**	**0.009**
	(/mm^3^)	14.8	(16.7)	9.7	(11.9)	3.6	(3.0)	**0.016**	**0.011**
CD27^+^ CD28^+^	(% CD8)	60.4	(24.1)	40.4	(23.7)	48.5	(26.0)	**0.037**	**0.026**
	(/mm^3^)	104.3	(51.4)	75.1	(57.0)	48.3	(31.9)	**0.010**	**0.095**

The CD8-senescence associated parameters were compared between the 3 groups (i) group 1 (non-severe COVID-19), (ii) group 2 (non-critical COVID-19) and (iii) group 3 (critical COVID-19). For CD8^+^ T cells subpopulation, results are presented in percentage among CD8 or in absolute number. When the difference between severity groups 1, 2 and 3 is significant, the parameter is in bold (*p* = or < 0.05) or italic bold when the difference is absent after adjustment on age (adjusted *p* value). Comparisons were tested using a one-way ANOVA. *P* value is from one-way ANOVA (univariate *p* value) and adjusted on age (adjusted *p* value)

#### *Myelo-senescence profile (*Fig. 2* and *Table S3*)*

**Fig. 2:**
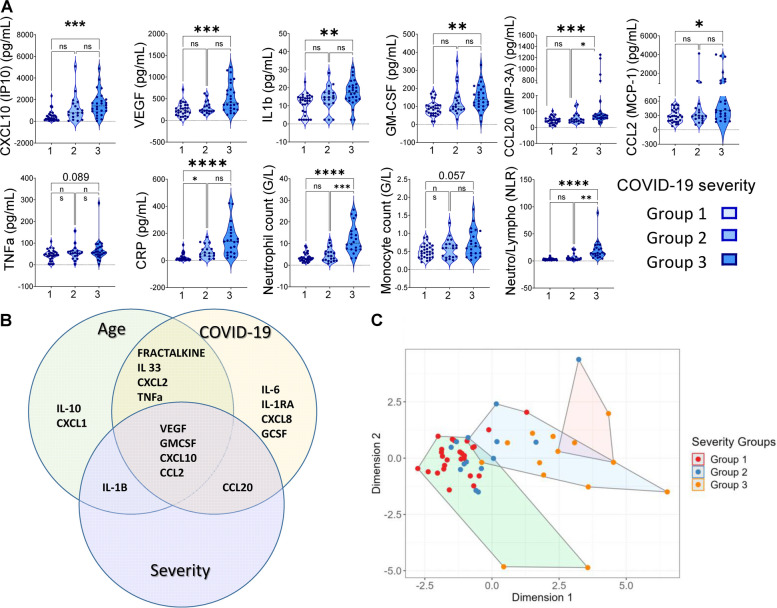
Myelo-senescence parameters positively associated with COVID-19 severity. A) CXCL10 (IP-10), VEGF, IL-1b, GM-CSF, MIP-3a, CCL2 (MCP-1), TNFa concentrations (pg/mL), CRP, neutrophil and monocyte counts and Neutrophil to Lymphocyte Ratio (NLR) were compared between severity groups. Two-group differences were tested using a two-sided unpaired t-test. The difference was considered as significant when the *p* value was equal to or under 0.05. The *p* value are summarized with asterisks (< 0.05*, < 0.01**, < 0.001***, < 0.0001****). B) Venn diagram showing the cytokines positively associated with: (i) COVID-19 (COVID-19^+^ *versus* COVID-19^−^), (ii) age in COVID-19^+^ patients (older *versus* younger cohort) and (iii) COVID-19 severity in older people. CXCL10, VEGF, GM-CSF, and CCL2 (MCP-1) are positively associated with age, COVID-19 and severity (results from Venny 2.1.). C) A clustering model (K-means) integrating the relevant senescence variable in a principal component analysis defined 3 clusters. Each point represents an individual for whom we have all the data for the variables listed (CXCL10, VEGF, IL-1B, GM-CSF, CCL2, CRP, neutrophils, monocytes, lymphocytes, NLR). The critical group 3 patients are mainly in the same clusters (red and blue) that is different from the green cluster in which non-severe non-critical patients are dominant

CRP, CXCL10, VEGF, GM-CSF, IL-1β, neutrophil counts and NLR showed a strong positive association with increased severity, both with and without adjustment for age. TNFα, CCL20, CCL2 and monocyte counts were weakly associated with increased severity after adjustment for age. G-CSF, IL-6, IL-1Ra and IL-10 were not associated with severity both with and without adjustment for age.

#### Immune ageing signature of COVID-19 severity in the elderly

The cytokines positively associated with ageing, COVID-19 and severity are presented in Fig. 2B. The Venn diagram shows that TNFα, VEGF, GM-CSF, CXCL10 and CCL2 were significantly positively associated with senescence, COVID-19 and severity.

We performed a K means clustering based on variables chosen for their clinical, biological and statistical significance, from which three clusters were identified. The majority of the severity group 1 (non-severe) patients were in the same cluster which was well separated from the critical patients group 3 cluster (Fig. 2C). The severity group 3 cluster displayed a high myelo-senescence signature.

**Fig. 3:**
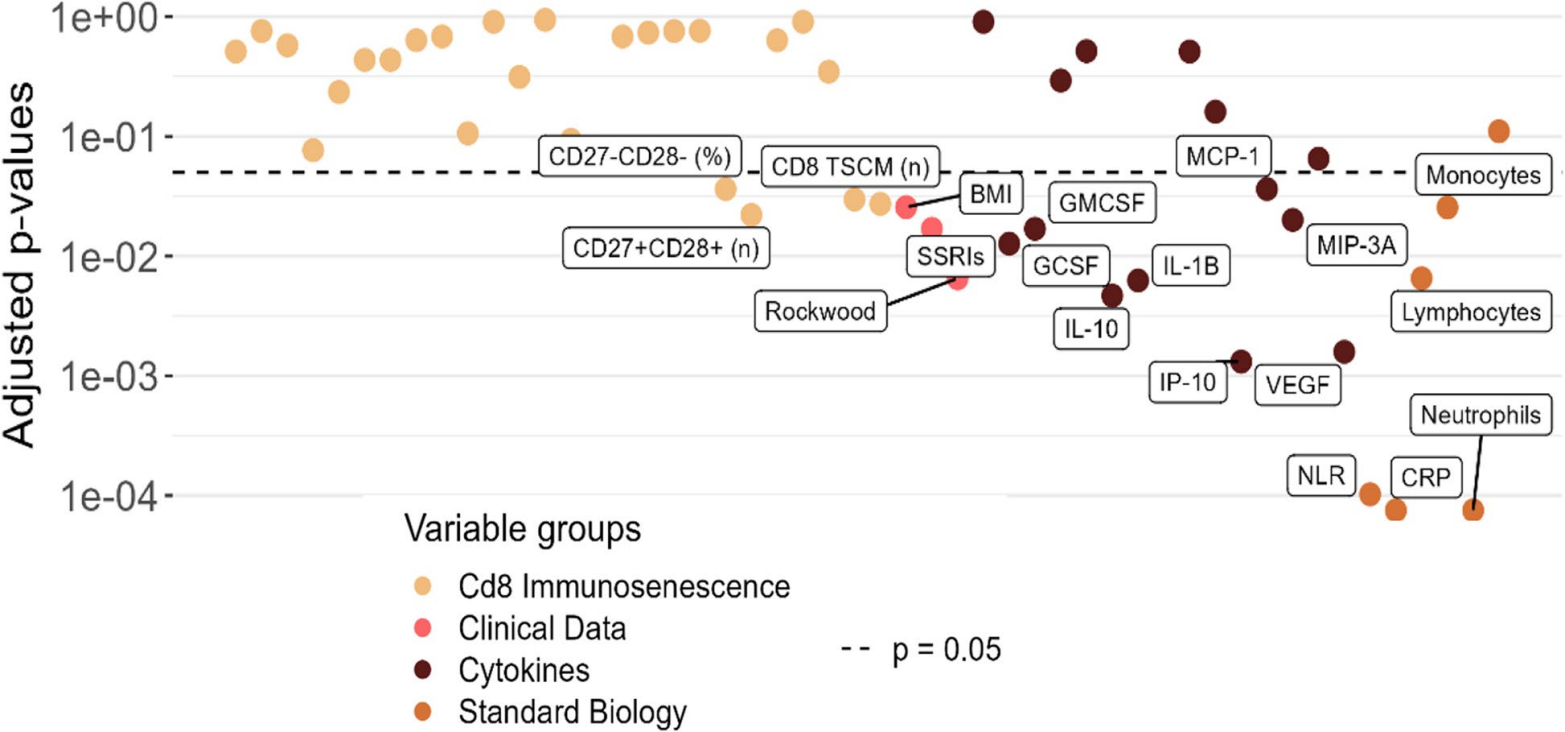
Immune ageing parameters positively associated with COVID-19 severity after correction for multiple testing. After FDR correction of *p* values for multiple testing, neutrophil, lymphocyte, monocyte count, NLR, CRP, VEGF, CXCL10, IL-10, IL-1B, G-CSF, GM-CSF, CCL2, CD27^+^CD28^+^, CD27^−^CD28^−^, TSCM-like CD8 + T cells, BMI (Body Mass Index), serotonin reuptake inhibitor (SRI) and Rockwood score were associated with severity. Bivariate analysis are adjusted for age

After FDR correction for multiple testing, the factors that remained positively associated with severity were the neutrophil counts, CRP, NLR, VEGF, CXCL10, GM-CSF, IL-1β, CCL2 and monocyte count, CD27^−^CD28^−^ among CD8^+^ T cell and clinical variables BMI, serotonin reuptake inhibitors, male sex and the Rockwood Frailty Index. CD27^+^CD28^+^ and TSCM percentage among CD8 lymphocytes remained associated with decreased severity (Fig. 3).

## Discussion

Older age is associated with a significantly higher risk of severe COVID-19 leading to increased mortality. However, immune parameters associated with COVID-19 in older patients are poorly described.

To address this, we performed an observational study in which we characterized CD8^+^ T cell phenotypes and cytokines associated with senescence, during COVID-19, in a cohort of older patients. We compared a COVID-19 positive cohort with an uninfected control cohort. We then investigated the factors associated with COVID-19 severity between groups of increasing severity from group 1 to 3. We found that a high level of the senescence-associated cytokines GM-CSF, CXCL10, VEGF and CCL2, NLR, and a high CD27^−^CD28^−^ percentage among CD8^+^ T cells, were positively associated with increased COVID-19 severity. CD8^+^ stem memory T cells (TSCM) and CD27^+^CD28^+^ CD8^+^ T cell were negatively associated with severity.

We found that gender, BMI and serotonin reuptake inhibitor treatment were positively associated with more severe COVID-19 disease as previously described [31]. In our cohort, comorbidities such as cardiovascular disease, chronic lung infection and diabetes were not associated with COVID-19 severity. Accordingly, the Charlson Comorbidity Index was not a risk factor for severe forms of COVID-19. Surprisingly, the COVID-19^+^ critical severity group (group 3) was younger than the other groups (groups 1 and 2). Thus, in very old people, the association between age and severity may no longer be linear. These observations are in line with the few existing reports on older patients cohort or sub-group analysis [2, 32].

We found that our COVID-19^+^ cohort had higher neutrophil counts, NLR, monocyte counts, CRP levels and a lower CD8 count compared to the control cohort, which is consistent with existing data [13, 19]. In our study, these parameters positively correlated with COVID-19 severity. The peripheral CD8^+^ T cell lymphopenia may reflect CD8^+^ T cell migration to the lungs. The lymphopenia in CM CD8^+^ cells appears to be particularly pronounced, as the difference was found in both count and percentage, suggesting that this was not solely a reflection of the global lymphopenia, in contrast to what was observed for EM and naïve cells.

The CD8^+^ T cell senescence parameters we examined (CD27^−^CD28^−^, EMRA, CD57, KLRG1, CD56% among CD8 and Sestrin-2 expression by CD8) were positively correlated together and defined a CD8 senescence profile. The NLR, known to be increased with age and associated with bad prognosis during infection [27], was positively correlated with this CD8 senescence profile whereas the CD4/CD8 ratio was inversely correlated. This is in concordance with previous findings showing that, in older people, CD4/CD8 ratio inferior to 1 is an immune risk phenotype for lung nosocomial infection [33]. Interestingly, we found that the CD8 senescence profile was inversely associated with early differentiated CD27^+^CD28^+^ and TSCM-like CD8^+^ T cells.

CD27^−^CD28^−^, CD57 and EMRA frequencies among CD8^+^ T cells were increased in the higher severity groups. This difference persisted for CD27^−^CD28^−^ after correction for multiple testing. To note, the comparison of absolute values did not reveal any difference between the groups, suggesting an over-occupation of the CD8 pool by senescent T cells in the group 3 that is particularly lymphopenic. Even if the statistical comparison could not be performed, the EMRA frequency among CD8^+^ T cell was increased in our older COVID 19^+^ cohort compared to a younger COVID 19^+^ cohort described in the literature (30.3% ± 23.4 in the geriatrics *versus* 20.0% ± 18.4 in younger adults, from Divij Mathew dataset) [34]. We observed increased expression of Sestrin-2 in the COVID-19^+^ cohort compared to the control cohort. This elevation could be associated with acute infection and cellular stress, which is known to upregulate Sestrin-2 expression [12]. Additionally, Sestrin-2 expression was found to be higher in CD8^+^ T cells from patients in severity group-2. However, given that the group 2 patients were older than those in the other groups, the lack of difference after age adjustment suggests that this may be attributed to increased expression with age, even within a cohort of patients over 70 years old.

Interestingly, the percentage and number of TSCM-like CD8^+^ T cells, which are early differentiated cells with a great self-renewal and differentiation potential after antigen exposure [35], was highly associated with a lower severity, even after FDR correction for multiple testing. The fact that the difference was strongly observed in percentage and in absolute value, despite comparable CD8 lymphocyte number between the groups 1 and 2, suggests that it is not a consequence of global lymphopenia. Furthermore, the numbers of EM, EMRA and CM were comparable between the severity groups, suggesting that they occupy a consistent position within the CD8 pool. Also, TSCM-like cells were more abundant in COVID-19^+^ group 1 (but not in groups 2 and 3) compared to the control group (data not shown). This suggests that CD8^+^ TSCM-like cells, even in low numbers, could proliferate early during in the course of the infection to provide protection against severe manifestations.

CXCL10, GM-CSF, VEGF, IL-1β and CCL2 were clearly positively associated with a higher COVID-19 severity in elderly patients even after adjustment for age and correction for multiple testing. In COVID-19^+^ patients, concentrations were all higher in our cohort compared to a younger cohort [14]. GM-CSF, VEGF, IL-1β and CCL2 were previously associated with ageing [16, 17, 29, 30] and our study confirms their association with senescence. In a clustering model (Principal Component Analysis), the severity group 3 appeared mostly grouped within one single cluster based on cytokine concentrations, confirming their importance in the development of severe infection. Accordingly, some reports suggest that the inflammaging could contribute to the cytokine storm [36], which is involved in the disease severity.

CXCL10 had previously been associated with frailty in older population [37, 38] We found in our study that CXCL10 could be an interesting marker of COVID-19 severity and also seems to be associated with ageing. Indeed, CXCL10 levels were higher in our older cohort compared to a younger one in which it was not associated with severity [14]. This suggests that it could be a marker of inflammaging with prognostic value in older patients. Interestingly, it negatively correlated with CD8^+^ T cell specific response in acute cases [1].

GM-CSF could contribute to the tissue infiltration by inflammatory myeloid cells described in moderate-to-severe forms of COVID19 [39]. We found in our study that GM-CSF concentration was higher compared to the younger cohort and positively associated with COVID-19 severity. Smith et al. study [14] reported that GM-CSF was not associated with severity in a younger cohort. Therefore, our results suggest a specific prognostic value in older patients. Accordingly, in an adult cohort, Thwaistes et al. found that GM-CSF could be a differential mediator of COVID-19 severity compared to flu severity particularly in patients over 70 years old [40]. Interestingly, a recent study examined the role of GM-CSF antagonism for COVID-19 care. Although no role was found in the whole population in a phase II clinical trial, the antagonism led to a clinical improvement (mortality and time to recovery from respiratory failure) in the sub-group of patients over 70 years old [41].

Single-cell analysis of bronchoalveolar lavages from critical COVID-19 patients revealed the abundance of inflammatory IL-1β-secreting myeloid cells, which could be involved in lung damages [42]. Indeed, NLRP3 inflammasome activation, which induces IL-1β, has been described in neutrophils from severe COVID-19 patients and this pathway seems to be particularly involved in older patients [27]. In a younger adults cohort (aged 53 to 72 years old), Del Valle et al. found that IL-1β had a low predictive value of COVID-19 mortality [43]. This suggests that IL-1β association with severity is age-specific.

During severe COVID-19, CCL2 secretion in the lung likely participates to unconventional monocyte lung infiltration linked with pathogenesis [18]. This mechanism was previously observed in aged skin where CCL2 over-secretion by senescent fibroblast decreased memory T cell activation and proliferation [44].

Our study has some limitations. First, we studied a real-life cohort and this study was exploratory in nature, thus the development of a statistical model with adjustment for variables of interest such as age, BMI, sex ratio, comorbidities or where patients live (in patient *versus* out patient) was not possible due to the low number of subjects in the cohort. Second, some socio-demographic criteria, such as ethnic origin, would have been a relevant criteria to include in the statistical analysis, but due to a lack of official data collection this could not be included in the model. However, we assume that the likely impact of this demographic parameter in our study would be minor. Finally, the size of the control cohort is not comparable to the size of the cohort of interest but, we assume that comparison with a larger cohort would have confirmed our key findings. However, our study has strengths to highlight. In the literature, cohorts of older people with significant size are rare. To our knowledge, no study of immunological ageing in COVID-19^+^ older people cohort is available. Our study has the advantage of being a prospective single-centre study, with a homogeneous inclusion and sampling time point, which limits certain biases. In our statistical approach, all results were adjusted for age, and strongest results were confirmed by correcting for multiple testing.(FDR). We believe that the findings of our study, highlighting the association with the severity of COVID-19 and the high levels of GMCSF and CXCL10 and low levels of CD8^+^ TSCM, require further investigation and confirmation on a larger scale.

## Conclusions

Our study is original in that it characterizes the ageing of the immune system and its association with COVID19 severity in 104 older patients over 70 years old. To summarize, we demonstrated that CD27^−^CD28^−^ CD8^+^ T cells, CRP, NLR, VEGF, CXCL10, GM-CSF, IL-1β and CCL2 are positively associated with the severity of COVID-19 disease in older patients. These observations are in accordance with previous studies conducted in younger patients [45]. This is the first study reporting the significance of GM-CSF, CXCL10 and CD27^−^CD28^−^ CD8^+^ T cells in the severity of COVID-19 specifically in an older population. These parameters, linked with senescence and myelopoiesis could be of clinical importance in the setting of infections such as COVID-19, especially in older patients. Our results also suggest that, in older patients, early precursor CD27^+^CD28^+^ and TSCM CD8^+^ T cells are protective from severe forms. Hence, our study provides keys for a better understanding of the underlying mechanisms linked with immune ageing in the COVID-19 severity, although further studies are needed to investigate these results in greater depth.

### Supplementary Information

**Supplementary Material 1**.

## Data Availability

The data supporting the findings of this study are available from the corresponding authors upon written reasonable request. The external validation cohort dataset [14] is publicly available at https://doi.org/10.1038/s41590-021-01028-7.

## Abbreviations

BMI: Body Mass Index
CM: Central memory
CRP: C reactive protein
EM: Effector memory
HFOT: High flow oxygen therapy
ICU: Intensive care units
IMV: Invasive Mechanical Ventilation
NK: Natural killer
NLR: Neutrophil to Lymphocyte Ratio
MFI: Mean fluorescence intensity
NIV: Non-Invasive Ventilation
nMFI: Normalized mean fluorescence intensity
PCA: Principal Component Analysis
qSOFA: Quick Sequential Organ Failure Assessment
SASP: Senescence-Associated Secretory Phenotype
TSCM: T stem cell-like memory cells
