# Supplementary material for: Increased levels of GM-CSF and CXCL10 and low CD8+ memory stem T Cell count are markers of immunosenescence and severe COVID-19 in older people
Source: Immun Ageing. 2024 May 7;21:28. doi: 10.1186/s12979-024-00430-7 (PMC11075216; doi:10.1186/s12979-024-00430-7)
Supplement: Supplementary file 1 — Supplementary Material 1. [file 12979_2024_430_MOESM1_ESM.docx]

**Supplementary tables**

**Table S1: CD8^+^ T cell differentiation subsets in the control and COVID-19^+^ groups**

Only CM were present in lower percentages and absolute numbers in the COVID-19^+^ cohort compared to the control cohort, whereas naïves, TSCM, EM, and EMRA were present in comparable percentages. Lower numbers of EM and naïve T cells may be the result of the global CD8 lymphopenia. Univariate *p* value is from two-sided unpaired t-test.

| Mean (SD) |  | Controls (N=21) | COVID-19^+^ (N=58) | p value | Group 1 (N=24) | Group 2 (N=14) | Group 3 (N=20) | *p*.value |
| --- | --- | --- | --- | --- | --- | --- | --- | --- |
| CD8^+^ T cells | /mm^3^ | 389.4 (312.1) | 225.1 (102.3) | **<0.0001** | 198.8 (132.9) | 212.4 (201.0) | 99.9 (73.6) | **0.006** |
| Naives | %/CD8 | 34.1 (18.4) | 36.87 (17.6) | ns | 38.8 (16.0) | 32.4 (20.0) | 37.7 (18.1) | ns |
|  | /mm^3^ | 100 (64.2) | 65.7 (14.8) | **0.0034** | 70.1 (34.6) | 53.9 (54.6) | 39.0 (34.7) | **0.045** |
| TSCM | %/CD8 | 3.9 (3.1) | 6.18 (5.5) | ns | 8.6 (7.0) | 5.6 (3.8) | 3.7 (2.6) | **0.009** |
|  | /mm^3^ | 9.3 (5.1) | 9.3 (6.3) | ns | 14.8 (16.7) | 9.7 (11.9) | 3.6 (3.0) | **0.016** |
| EM | %/CD8 | 5.3 (4.5) | 4.34 (4.7) | ns | 3.8 (4.53) | 5.9 (6.0) | 3.88 (3.6) | ns |
|  | /mm^3^ | 21.3 (23.7) | 15.4 (20.8) | **0.0019** | 9.7 (16.5) | 25.0 (55.4) | 5.8 (7.6) | ns |
| CM | %/CD8 | 26.3 (15.9) | 19.1 (14.0) | **0.056** | 20.82 (14.25) | 17.4 (14.4) | 18.22 (13.8) | ns |
|  | /mm^3^ | 78 (57.8) | 45.7 (14.2) | **<0.0001** | 42.9 (42.5) | 41.7 (56) | 20.2 (27.1) | ns |
| EMRA | %/CD8 | 30.3 (24.0) | 33.5 (23.8) | ns | 26.2 (21.7) | 37.6 (21.8) | 37.2 ( 25.3) | ns |
|  | /mm^3^ | 141.1 (167.1) | 80.3 (56.8) | ns | 62.6 (83.2) | 81.3 (76.2) | 36.2 (30.7) | ns |

**Table S2: cytokines in older people: COVID-19^+^ *versus* COVID-19 negative control cohort**

The majority of the senescence associated cytokines were significantly in higher concentration in the COVID-19^+^ geriatric cohort. The difference was not significant for IL-10, CXCL8, CXCL1 and IL-1B concentration. Results are presented in mean (standard deviation). *P* value is from two-sided unpaired t-test. The difference was considered as significant when the *p* value was under 0.05.

| Variable in mean (SD) pg/mL | control cohort | COVID-19+ cohort | *p* value |
| --- | --- | --- | --- |
| Inflammageing and Senescence associated secretory phenotype (SASP) | | | |
| **plasma TNFa** | 14 (14) | 56 (37) | <0.0001 |
| **plasma IL-6** | 72 (282) | 543 (3941) | <0.0001 |
| **plasma VEGF** | 222 (130) | 352 (252) | 0.0014 |
| **plasma IL-1RA** | 1554 (1775) | 4166 (7496) | 0.0055 |
| **plasma GMCSF** | 73 (47) | 118 (66) | 0.0005 |
| **plasma GCSF** | 50 (22) | 135 (506) | <0.0001 |
| **plasma CCL2** | 237 (94) | 561 (824) | 0.0009 |
| **plasma CCL20** | 41 (30) | 108 (222) | 0.016 |
| **plasma CXCL2** | 158 (146) | 286 (383) | 0.0182 |
| plasma CXCL1 | 124 (49) | 148 (76) | ns |
| plasma CXCL8 | 7.9 (6.1) | 19 (50) | ns |
| plasma IL-1B | 13 (5.6) | 14 (6.7) | ns |
| plasma IL-10 | 485 (1898) | 347 (512) | ns |
| Myeloid lineage | | | |
| **plasma CXCL10** | 146 (98) | 978 (1047) | <0.0001 |
| **plasma FRACTALKINE** | 943 (376) | 1894 (2622) | 0.0027 |
| **plasma IL-33** | 24 (9.3) | 36 (44) | 0.0294 |

**Table S3: cytokine correlation with COVID-19 severity in older people**

Plasma CXCL10, VEGF, GM-CSF, IL-1b were highly associated with increased severity (p<0.005) after adjustment on age. TNFa, MIP-3a and CCL2 were weakly associated with increased severity. *P* value is from one-way ANOVA (univariate p value) and adjusted on age (adjusted p value). The difference was considered as significant when the *p* value was egal or under 0.05.

| Variable pg/mL mean (SD) | Group 1 | Group 2 | Group 3 | univariate *p*.value | adjusted *p*.value |
| --- | --- | --- | --- | --- | --- |
| **plasma TNFa** | 44.2 | 56.1 | 67.8 | 0.071 | 0.043* |
|  | (24.6) | (32.5) | (51.0) |  |  |
| **plasma VEGF** | 241.7 | 296.2 | 536.3 | <0.001*** | <0.001*** |
|  | (148.6) | (139.1) | (330.0) |  |  |
| **plasma GMCSF** | 89.4 | 129.4 | 149.4 | 0.002** | 0.004** |
|  | (41.7) | (79.5) | (68.9) |  |  |
| **plasma IL-1B** | 11.0 | 14.1 | 17.3 | 0.002** | 0.001** |
|  | (5.6) | (6.7) | (7.0) |  |  |
| **plasma CXCL10** | 467.0 | 1157.1 | 1611.5 | <0.001*** | <0.001*** |
|  | (495.9) | (1195.2) | (1139.7) |  |  |
| plasma FRACTALKINE | 2114.7 | 1368.7 | 2199.2 | 0.557 | 0.805 |
|  | (4158.5) | (624.0) | (1257.6) |  |  |
| plasma IL 33 | 42.8 | 29.1 | 35.4 | 0.589 | 0.608 |
|  | (70.7) | (11.3) | (13.3) |  |  |
| **plasma CCL2** | 291.8 | 572.6 | 947.0 | 0.017* | 0.020* |
|  | (133.6) | (903.7) | (1175.6) |  |  |
| plasma IL-6 | 26.6 | 146.8 | 1547.2 | 0.336 | 0.173 |
|  | (18.7) | (458.2) | (6928.0) |  |  |
| plasma IL-1RA | 1870.6 | 3919.9 | 7394.9 | 0.028* | 0.077 |
|  | (1133.5) | (4315.2) | (12001.0) |  |  |
| **plasma CCL20** | 45.1 | 57.3 | 217.0 | 0.008** | 0.051 |
|  | (22.4) | (33.2) | (363.6) |  |  |
| plasma CXCL8 | 9.0 | 15.4 | 35.5 | 0.149 | 0.364 |
|  | (7.0) | (16.9) | (85.5) |  |  |
| plasma GCSF | 53.5 | 77.0 | 288.0 | 0.209 | 0.116 |
|  | (28.8) | (42.9) | (884.0) |  |  |
| plasma IL-10 | 204.9 | 296.6 | 583.3 | 0.022* | 0.092 |
|  | (129.9) | (145.5) | (844.5) |  |  |
| plasma CXCL1 | 160.3 | 153.0 | 133.5 | 0.434 | 0.371 |
|  | (104.3) | (69.1) | (39.5) |  |  |
| plasma CXCL2 | 368.9 | 216.6 | 255.5 | 0.365 | 0.274 |
|  | (595.7) | (107.0) | (175.3) |  |  |

**Table S4:** antibodies used for cytometry stainings

| **Target** | **Fluorochrome** | **Provider** | **Clone** | **Reference** |
| --- | --- | --- | --- | --- |
| CD3 | BV510 | BD | UCHT1 | 563109 |
| CD8 | BB515 | BD | RPA-T8 | 564526 |
| CD4 | PC7 | Beckman Coulter | SFCI12T4D11 | 6607101 |
| CD45RA | BV421 | Biolegend | HI100 | 304130 |
| CD27 | PE/CF594 | BD | M-T271 | 562297 |
| CD28 | PC5 | BD | CD28.2 | 555730 |
| CD95 | PE | BD | DX2 | 555647 |
| CD56 | APC-R700 | BD | NCAM16-2 | 565139 |
| CD57 | PE/CF594 | BD | NK-1 | 562488 |
| NKG2A | BV421 | BD | 131411 | 747924 |
| KLRG1 | AF488 | ThermoFisher | 13F12F2 | 53948842 |
| DAP12 | AF647 | BD | 406288 | 566603 |
| Ki67 | PE | BD | RUO | 556027 |
| Sestrin-2 | AF700 | CST | D1B6 | 8487 |

**Supplementary Figures**

104 hospitalized patients

>70 yo included between 24/04/2020 and 19/03/2021

Confirmed COVID-19

n=81 (ICU n=27, non CIU n=54)

Control

n=23 COVID-19 negative

**Group 1**: Non severe COVID-19

n=32

**Group 2**: Non-critical

COVID-19

n=20

**Group 3**: Critical COVID-19

n=29

**Figure S1: Flow chart illustrating the study design**


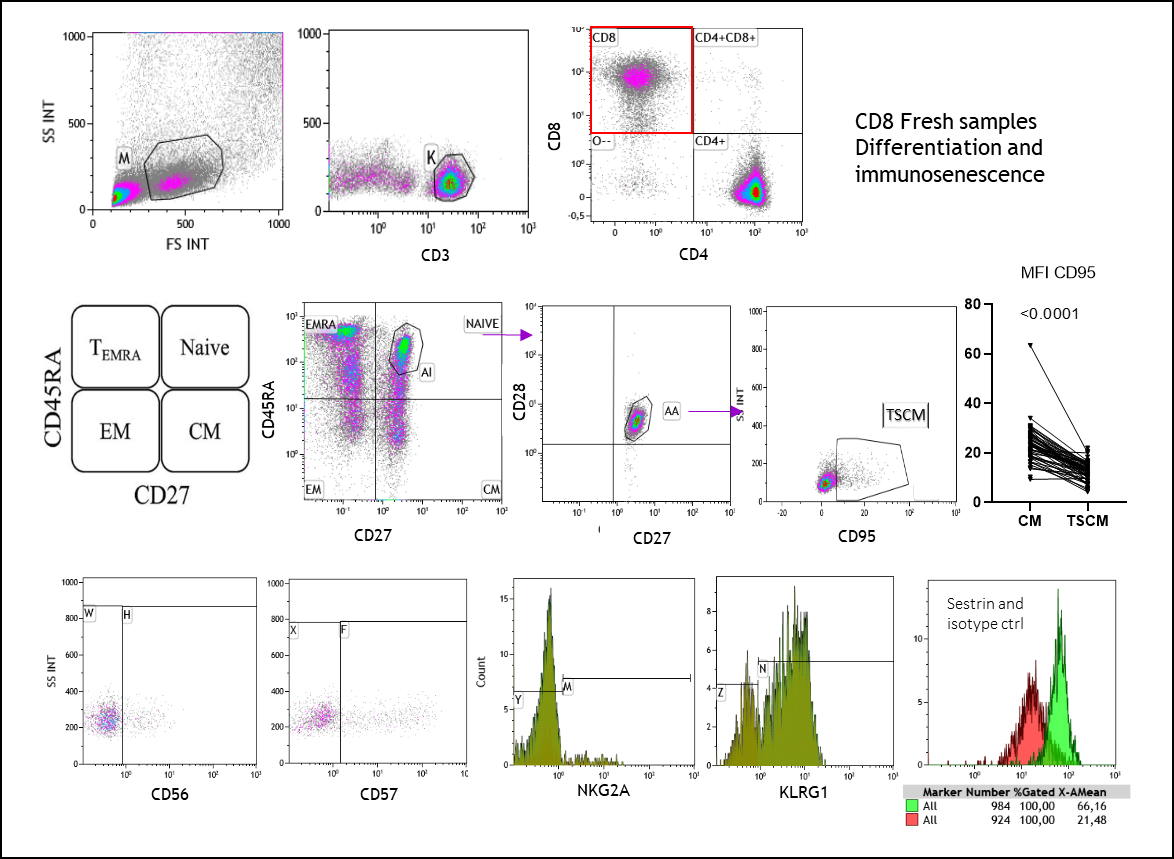


**Figure S2: Gating strategy for CD8 T cell immunosenescence characterization**. T cell phenotyping for immunosenescence markers was done prospectively on fresh blood samples. The gating strategy consists in CD3+ CD8+ CD4-negative lymphocyte selection. The naïve, CM, EM and EMRA differentiation stages were discriminated using CD45RA and CD27 quadrant and CD28 positivity for naïve cells. The stem cell memory (TSCM)-like CD8 T cells were considered as the naïves expressing CD95. The CD95 level of expression in central memory (CM) was higher compared to the TSCM-like. The NK-receptors CD56, CD57, NKG2a and KLRG1 percentages were evaluated thanks to dot plots or histograms. Sestrin-2 expression was interpreted in normalized MFI according to its isotype control (histograms).

**
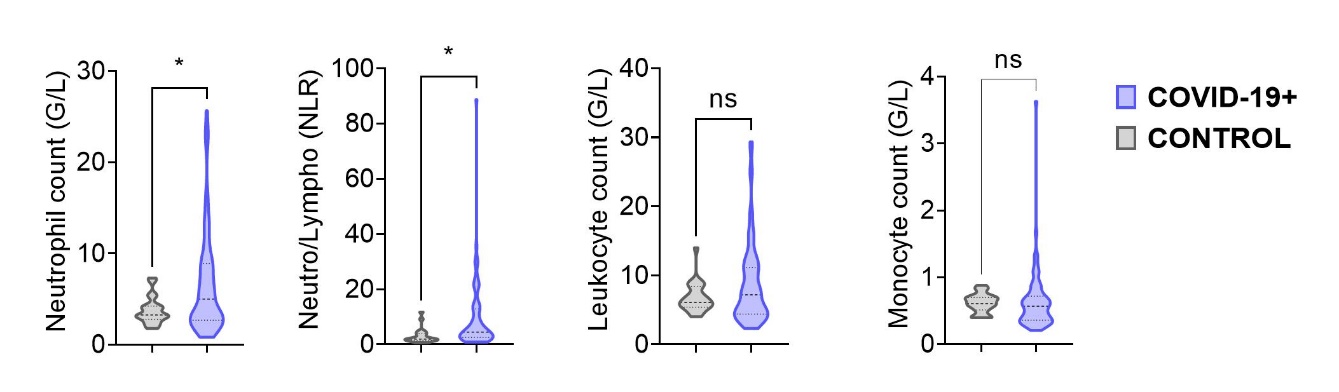
**

**Figure S3 Myeloid cells associated with COVID-19 infection**

The COVID-19^+^ cohort (blue violin plot) was compared with the control cohort (grey violin plot) for leukocyte, neutrophil, monocyte counts and Neutrophil to lymphocyte ratio. Two-group differences were tested using a two-sided unpaired t-test. The difference was considered as significant when the *p* value was equal to or under 0.05. The *p* value are summarized with asterisks (<0.05*, <0.01**, <0.001***, <0.0001****).

**Figure S4 Immunosenescence profile of the cytokines associated with senescence in the COVID-19 cohort.** CRP, NLR, neutrophil count, IL-6, MIP-3A, VEGF, CCL2, G-CSF, TNFα, IL-10, IL-1β, IL-1RA, GM-CSF, CXCL10 and monocyte count are clustered and could define a myeloïde-senescence profile (surrounded in red). The numbers indicate the R coefficient of Pearson correlation.

**
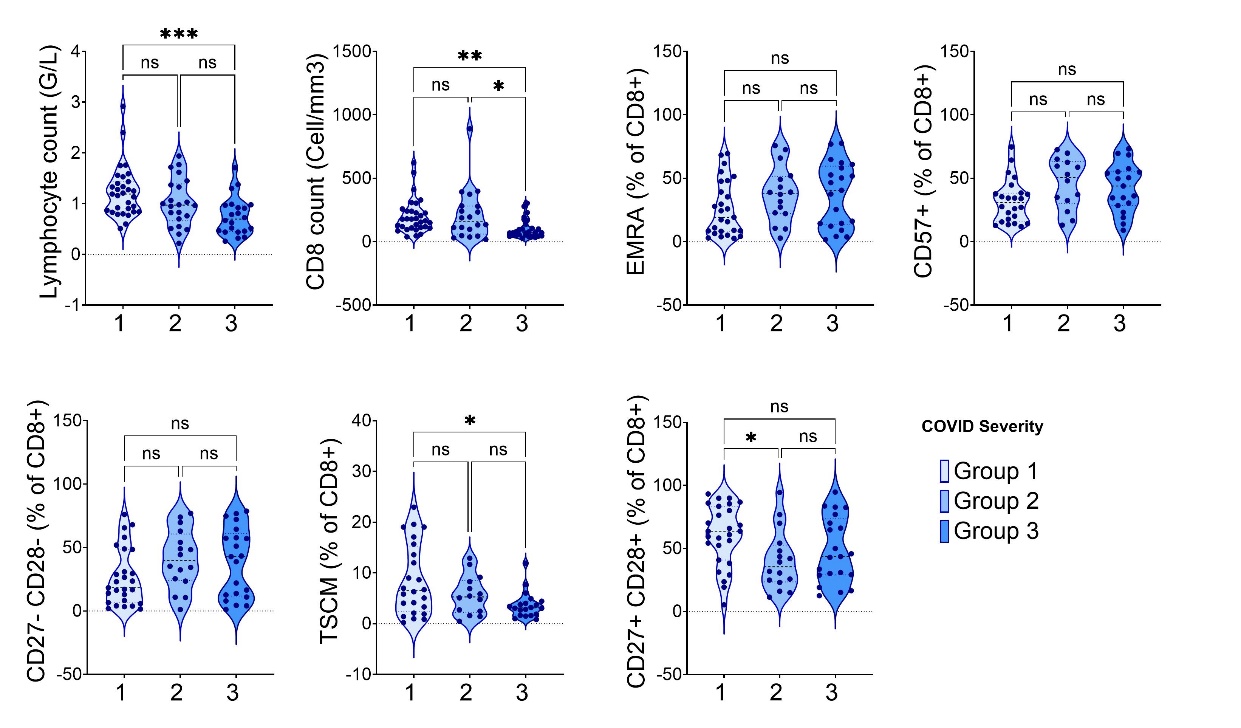
**

**Figure S5 CD8^+^ T cell subsets association with COVID-19 severity**

CD8^+^ T cell subsets were compared between severity groups. Two-group differences were tested using a two-sided unpaired t-test. The difference was considered as significant when the *p* value was equal to or under 0.05. The *p* value are summarized with asterisks (<0.05*, <0.01**, <0.001***, <0.0001****).
